# Beliefs as Self-Sustaining Networks: Drawing Parallels Between Networks of Ecosystems and Adults’ Predictions

**DOI:** 10.3389/fpsyg.2015.01723

**Published:** 2015-11-12

**Authors:** Ramon D. Castillo, Heidi Kloos, Michael J. Richardson, Talia Waltzer

**Affiliations:** ^1^Facultad de Psicología, Universidad de TalcaTalca, Chile; ^2^Center for Cognition, Action, and Perception, Department of Psychology, University of Cincinnati, CincinnatiOH, USA; ^3^Department of Psychology and Center for Cognitive Science, Rutgers University, New BrunswickNJ, USA

**Keywords:** information theory, average mutual information, uncertainty, degree of order, predictive learning

## Abstract

In this paper, we argue that beliefs share common properties with the self-sustaining networks of complex systems. Matching experiences are said to couple with each other into a mutually reinforcing network. The goal of the current paper is to spell out and develop these ideas, using our understanding of ecosystems as a guide. In Part 1 of the paper, we provide theoretical considerations relevant to this new conceptualization of beliefs, including the theoretical overlap between energy and meaning. In Part 2, we discuss the implications of this new conceptualization on our understanding of belief emergence and belief change. Finally, in Part 3, we provide an analytical mapping between beliefs and the self-sustaining networks of ecosystems, namely by applying to behavioral data a measure developed for ecosystem networks. Specifically, average accuracies were subjected to analyses of uncertainty (H) and average mutual information. The ratio between these two values yields degree of order, a measure of how organized the self-sustained network is. Degree of order was tracked over time and compared to the amount of explained variance returned by a categorical non-linear principal components analysis. Finding high correspondence between the two measures of order, together with the theoretical groundwork discussed in Parts 1 and 2, lends preliminary validity to our theory that beliefs have important similarities to the structural characteristics of self-sustaining networks.

## Introduction

Which object will get to the bottom of a water tank the fastest? When presented with this question, one might want to make a wild guess, independently of any guesses made before. More likely though, one will employ a specific belief, one that ties together previous experiences about sinking objects, to allow for systematic predictions. One might predict, for example, that the heavier object will sink faster, independently of its size, shape, shading, or texture. Such beliefs are necessary for adaptive functioning: they guide our attention to certain aspects of the context, and they allow us to anticipate the future. However, our understanding of the context in which beliefs emerge and change remains incomplete. For example, people have everyday experiences with water and objects sinking in it. Why, then, do we still have mistaken beliefs about sinking objects, often ignoring the relevant feature of material density when making predictions?

The goal of the current paper is to offer a new way of conceptualizing beliefs, including mistaken beliefs, one that could shed light on both their emergence and their stability over time. The specific argument is that beliefs are structured and organized by means of a self-sustaining network similar to that which characterizes many complex systems, including ecosystems. The components of this network are individual experiences, similar enough to become coupled with each other. It is through this coupling that the experiences become mutually reinforcing. Here, we seek to spell out the explanatory mechanisms of such coupling, using our understanding of ecosystems as a guide.

Our paper is organized as follows: in Part 1, we describe the analogy between beliefs and ecosystems, as a first illustration of a belief as a self-sustaining network. In Part 2, we then present the implications of this view, particularly as they relate to the emergence and change of beliefs. Finally, in Part 3, we provide a proof of concept for this proposal, namely by comparing two measures of order: one that was derived from complex ecosystem networks, and one that was derived from a traditional principal component analysis (PCA) that is used to identify and characterize underlying constructs from a covariance matrix.

### Beliefs as Self-Sustaining Networks of Experiences

Beliefs vary a great deal from each other. They range from trivial to fundamental in their scope, and they differ in the number of unique experiences they pertain to. Beliefs might be as narrow as the conviction that a reader understands these words, or they might be part of a larger system of beliefs, say that humans engage in meaningful activities to further their long-term goals. For simplicity, we will focus on single beliefs only, rather than groups of beliefs. However, it is likely that the same claims hold for different or nested scales of organization, whether we are referring to mundane details of everyday life or to fundamental principles of order.

Here, we propose that a belief is a network of perceptual experiences that have something in common. To explain, consider a context in which one has to predict the sinking behavior of objects. There are numerous features that vary in each display, including shading, spatial position, temperature, haptic sensations, odor, acoustic properties, as well as variation in heaviness, size, and shape of the objects. It is unlikely that we attend to all of these features equally. Instead, some features will have attentional priority, based on idiosyncratic factors of salience and perceived relevance. For example, the experience of heaviness, more so than the experience of size or shape, is likely to win out in salience, perhaps because heaviness, like sinking behavior, has a haptic component of downward force (cf. Kloos and Amazeen, 2002). Our argument is that similar experiences will enter a form of coupling, a coordination in which they mutually reinforce each other^1^. This coordination is the start of a belief.

**FIGURE 1:**
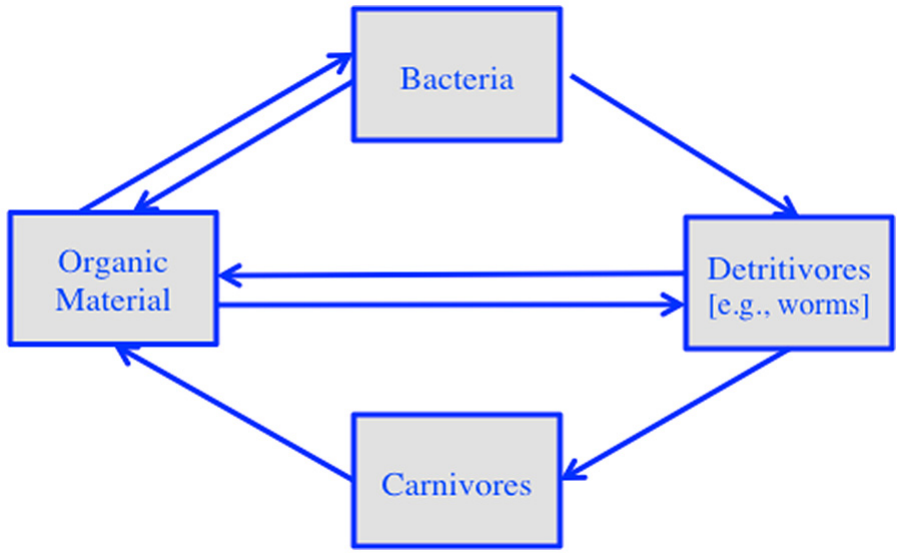
**Schema of an idealized ecosystem exchanging energy in a self-sustaining network**.

The metaphor of beliefs as networks of experiences is based on ideas derived from our understanding of complex systems, particularly ecosystems. To illustrate, consider a simplified ecosystem of the following components: organic material, bacteria, detritivores (e.g., worms), and carnivores (see **Figure 1**). Organic material provides the nutrition for bacteria and detritivores. Bacteria feed detritivores, which in turn feed carnivores. And both detritivores and carnivores turn into organic material, thus starting the cycle all over again. The individual species connect to each other through energy exchange, where one part of the network provides food for another part (and vice versa). The resulting network is self-sustaining, meaning that it can survive disruptions in external energy supply, for example during a drought (cf. Ulanowicz, 1986).

Just as components of an ecosystem are linked to each other on the basis of energy transfer, we claim that individual experiences are linked to each other on the basis of a transfer of *meaning*. Here, meaning is defined as a perceivable change in a feature that matters to the organism (i.e., relevant feature fluctuation). The fluctuation could be a change in light, sound, heat, or any other physical, chemical, or haptic property. For the feature change to be considered meaningful, two requirements have to be met: (1) the feature change needs to be perceivable, and (2) it has to have relevance in the context of the task. As such, meaning and energy share an important characteristic: they both can do work. While energy causes a change in general, meaning causes a specific kind of change: an action of the agent who seeks to solve a problem.

To develop the analogy between energy and meaning further, note that the energy in an ecosystem includes various forms of kinetic energy (e.g., heat energy, light energy) as well as potential energy (e.g., calories stored in individual organisms). Similarly, meaning can be available in ongoing experience (e.g., moment-to-moment fluctuations in structured light as one prepares an action); and it can be available in past experiences that are part of the network. When the relevant feature fluctuation in an experience matches that of a previous experience, the two get coupled. This is analogous to the coupling that takes place between two species that match in energy supply and demand, such as between detritivores and carnivores.

In an ecosystem, the potential energy stored in a component of the network (e.g., in a worm) gets transferred to another component (e.g., carnivore). While the transfer can be bi-directional, such as in the case of bacteria and organic material, there is a clear direction. A directional transfer of meaning, in contrast, might be rare. It might be restricted to cases that involve an inference or deduction from one experience to the next (e.g., hypothesis testing). In all other cases, including when making predictions about sinking objects, the transfer of meaning is likely to be bidirectional, not based on explicit inferences or deductions, but on the basis of a match. When two experiences have a feature variation in common (e.g., heaviness, from the example above), they get coupled on the basis of that commonality, yielding a kind of transfer that is analogous to synchrony (cf. Strogatz, 2003).

There are several interrelated concepts relevant to self-sustaining networks that could be of use to understanding the nature of beliefs. The first one pertains to *autocatalysis*, a specific form of positive feedback in which the effect of every successive connection in the network is strengthened over time (Kauffman, 1995). To use the ecosystem example again, positive feedback loops come in the form of energy flow, say from organic material to bacteria, to worms, and back to organic material. The resulting outcome is the recycling of energy, enough for creatures to sustain themselves despite interruptions in external energy supply. In the case of beliefs, meaning is the energy of the system, and the positive feedback loops come in the form of directed coupling of similar experiences. They bring about an increased similarity among experiences, consistent with the idea of a silent re-description of experiences, a sort of growth that takes place even in the absence of a task (Karmiloff-Smith, 1992; Spencer et al., 2009; see also Kounios and Beeman, 2014 for a similar claim on a neurological level).

The second concept of interest pertains to *circular causality*, the idea that components of the network, which give rise to the higher-order configuration, are constrained by the higher-order configuration itself (cf. e.g., Turvey, 1990, 2007; Kelso, 1995; Turvey and Carello, 1996; Smith, 2005; Laland et al., 2012). Species that are compatible in energy use seek to get coupled into a network that then affects the nature of the energy use among those species. Applied to beliefs, sufficiently similar experiences get coupled into a network that then affects the similarity among past experiences. It is this property of mutually reinforcing experiences that constrains what a person will remember about a prior experience. Whatever feature becomes coupled to another will now increase in salience during a subsequent event, by virtue of having been included in the self-sustaining network.

The third concept of interest pertains to *centripetality*, the idea that a network will attract resources into its circuit to sustain itself (Ulanowicz, 1986; Kauffman, 1995). That is to say, the network will draw in energy resources and eliminate competition over time. If two species seek to enter the same network, for example, the ecosystem will favor the one that provides the most efficient energy use. This is where the network obtains its character of agency, its inner life, driven by a force to sustain itself and grow. Applied to beliefs, centripetality is the force that draws in experiences that strengthen the whole, amplifying the relevance of confirming evidence and rendering conflicting evidence as irrelevant. It is by this feature that the system can stay stable even as individual experiences might get replaced.

Taken together, the principles of autocatalysis, circular causality, and centripetality give rise to a self-sustaining network, one that can sustain its order despite external fluctuations (cf. Juarrero, 1999). These principles can explain relevant aspects of beliefs, including their storage, retrieval, and apparent agency. For example, storage comes for free in a system that features autocatalytic processes. These are the processes that allow the system to amplify itself, without requiring uninterrupted external support. Furthermore, coupling can explain retrieval: when feature variability that are coupled with a component of belief are present in a task context, the belief will be retrieved. Centripetality bestows the network with agency, explaining why a prior belief affects new learning in such a way that it sustains itself. Finally, the idea of circular causality explains the dialectic character of beliefs to be tied to both new task contexts and to already existing experiences. What are the implications of this view for our understanding of how beliefs emerge and change?

### Implications for Explaining Emergence and Change

Questions about belief emergence and change have fueled theoretical advances in how to conceptualize beliefs. To what extent does our network theory of beliefs add to this conversation? In what follows, we seek to address this question, looking first at emergence, and then at change.

#### What Leads to the Emergence of Beliefs?

In recent years, emergence has begun to surface as an important topic of study (e.g., Oyama, 2000; Johnson, 2002; Deacon, 2011). Even so, the explanatory process that yields emergence is still rather nebulous. How could something entirely novel emerge, spontaneously, without being reduced to a flow chart of what came first, second, third, and so on? It is no surprise, then, that the idea of emergence has not yet fully shed its mystical character in the study of beliefs. Ohlsson (2011, p. 294) captures it as such: “The main triggering condition of the formation of a new belief is that new information knocks on the doors of perception and asks to be let in.” Even if we were to be able to define ‘information’ in this context, it would still leave the question open as to how it ‘gets in.’ The link we seek to draw between beliefs and ecosystems offers a theory of emergence that might dispel some of this mystery.

Note that the difficulty with the concept of emergence is not restricted to mental processes. In fact, it is seemingly in conflict with the universal tendency of global systems to move toward less structure, rather than more (cf. Gershenson and Fernandez, 2012). To address this conflict, a suggestion has been made that local order emerges for the purpose of moving the larger system toward equilibrium, faster than the system would move toward equilibrium otherwise (Odum, 1988; Swenson and Turvey, 1991). Put differently, the local self-organization is said to emerge precisely for the purpose of dissipating the disequilibrium that exists in the larger system.

To illustrate, consider the self-organization of convection cells in a fluid that is being heated from below (Pearson, 1958). The heat discrepancy between the bottom and the top of the fluid constitutes a disequilibrium that needs to be dissipated. If this heat gradient is low (i.e., heat levels below and above the fluid are similar), water molecules will move randomly, without giving rise to ordered convections. It is a certain heat gradient that gives rise to ordered convection cells. Thus, the emergence of structure is tied to the dissipation of a gradient, such that the law of maximal disorder is obeyed (i.e., the second law of thermodynamics).

The same concept can be applied to the emergence of life: in a simplified scenario, plants dissipate the gradient of light (among many other gradients), herbivores dissipate the gradient of energy stored in plants, and carnivores dissipate the gradient of energy stored in herbivores (Swenson and Turvey, 1991). Here, the relevant gradient refers to the off-and-on availability of food for the plants and animals that make up the ecosystem. The ecosystem dissipates this gradient through the coordination of energy exchange between species, and the network stores its own energy, in effect balancing out the energy gradient.

Our claim is that the same principle – the dissipation of a gradient – explains the emergence of beliefs. Rather than caloric energy, the relevant gradient pertains to meaning: the structured variability that makes it possible to complete a task. To illustrate this, consider the simple task of a predator catching its prey. The spatial relation between predator and prey defines meaning for the predator, an invariant property that needs to be established stably before the predator can catch its prey. If the predator gets distracted, say by a loud noise, the invariant property will be lost, at least momentarily. It is this interruption that creates the gradient of meaning. To retain meaning in the face of interruption (i.e., in the face of the meaning gradient), the network of coupled experiences comes into play. Thus, the intermittent nature of meaning availability results in a stable organization. It is the network of coupled experiences that bridges the meaning gradient, in effect dissipating the gradient. Put differently, it is the off-and-on availability of meaningful variability that brings about beliefs.

There is evidence that noise can lead to the emergence of a new organization in a cognitive system (Kitajo et al., 2003). Most recently, this principle has been demonstrated in a task in which participants had to guess an outcome after learning about the premises (Stephen et al., 2009a,b; Dixon et al., 2010). There were two strategies that could be used to solve this problem: one required a one-to-one coupling with the display (participants had to consider the behavior of each gear in a series of interlocked gears); the other one allowed participants to go above moment-to-moment details of the task and solve the problem by applying a generalized rule. Importantly, participants were faster to adopt this generalized rule when the display was moved randomly, in effect disrupting a one-to-one coupling with it.

Taken together, there are several conditions that need to be met before a belief emerges. First, there needs to be some relevant feature variability, variability that affords a successful completion of the task. Second, there has to be sufficient capability to couple with this variability. The coupling gives rise to an experience, a momentary snapshot of the event. Third, there needs to be a certain amount of noise to create a gradient in meaningful variability. This can come in the form of either structured or unstructured variability, not too much and not too little, to push for local coupling among experiences. Finally, experiences need to be similar enough to couple with each other. This coupling, the emergent belief unifying individual experiences, will amplify the similarity among experiences and filter out the noise. As such, it bridges the gradient in meaning.

#### What Does it Take to Change Beliefs?

So far, we have discussed beliefs under the premise that they are essential for adaptive functioning. They make prediction possible, they constrain the field of attention, and they bridge the interruption of relevant feature variation. There is another part to beliefs, however. Beliefs can be overly simplistic, incomplete, or misleading (for a review, see Vosniadou, 2008). And in this case, when there is a clash between reality and one’s belief, adaptive behavior can be hampered: predictions could be mistaken, attention could be focused on the wrong feature variation, and the feature variation that matters could go unnoticed. That is when the content of a belief needs to change. To what extent is this issue of mistaken beliefs in line with our network theory of beliefs?

Network theory indeed anticipates the presence of mistaken beliefs, precisely because of the attributes that are characteristic of self-sustaining networks. Individual experiences that give rise to beliefs are necessarily local, based on the presence of a feature variation that is salient in the moment in which it is perceived. Momentary salience does not necessarily mean long-term relevance, and the local focus does not ensure global veridicality. And yet, this initial experience sets in motion the emergence of a belief, following a bottom–up process that is biased toward replicating itself. It is this causal circularity that interferes with a rational reality check. And it is the autocatalysis that constrains subsequent experiences to amplify the ones that came before, without the luxury of a birds-eye view of correctness. It is no surprise, then, that mistaken beliefs emerge in domains in which local phenomenological experiences conflict with global scientific understanding (e.g., physical science, earth science).

Network theory also anticipates the fact that a change in belief can be challenging at times. To review, there is ample research suggesting that conflicting evidence is sometimes ignored (Ditto and Lopez, 1992; Munro, 2010); that conflicting evidence yields only superficial adjustments to a mistaken belief, leaving the main parts of the belief intact (e.g., Vosniadou and Brewer, 1992); and that confirming evidence is actively sought out (better known as a confirmation bias, e.g., Wason, 1960; Nickerson, 1998). If we consider beliefs as self-sustaining networks, built on the basis of local similarities, all of these findings follow naturally.

For example, given that a belief network serves the purpose of bridging interruptions in one kind of feature fluctuation, fluctuations that do not support the network must be ignored by design. In fact, such a latter feature fluctuation might in fact strengthen the existing belief (also known as the ‘backfire effect,’ cf. Lord et al., 1979; Nyhan and Reifler, 2010). This is because it could contribute to the size of the gradient that the network seeks to bridge. The tendency of a network to benefit from perturbation, known as *antifragility*, has also been documented in networks more generally (Taleb, 2012). This underscores the difficulty that is built into changing a belief network.

Given the forces of centripetality, networks do not change passively and incrementally (e.g., with each new experience). Instead, experiences are sought out actively, to maximally support the experiences at are already part of the network. Thus, the confirmation bias is not an abnormality of an otherwise rational mind. Instead, it is a direct result of the central properties of self-sustaining networks. Interestingly, the agency of networks is only in the service of the whole. Thus, local components can be added, removed, or changed as long as the whole is preserved. We have indeed found a certain degree of arbitrariness in local aspects of a belief, as long as the whole is preserved (cf. Kloos and Van Orden, 2012).

How, then, can the change of a mistaken belief be accomplished? Merely confronting mistaken beliefs with conflicting evidence is likely to fail, given the self-sustaining character of the network. For a change to take place in a network, the change must be beneficial to the overall network (cf. Ulanowicz, 1986, 1998, 2000). In the case of beliefs, such benefit is in the increased efficiency of coupled experiences. In other words, before they can be changed, beliefs have to lose their advantage faster than they can replenish internally, as part of the network (see also Ohlsson, 2011). Such changes would correspond to a non-linear or ‘catastrophic’ phase transition (bifurcation) analogous to when convection roles emerge a critical energy gradient.

In sum, our network theory of beliefs provides the first coherent framework of belief change, anticipating spontaneous, sudden, non-linear changes in belief structures, as well as both the presence of mistaken beliefs and the difficulty in changing mistaken beliefs. This approach to beliefs is different from existing theories of beliefs that have an empiricist or nativist bent: it does not put causal power exclusively in contextual factors, nor does it require structures to exist *a priori*. As such, it provides the perfect launching ground toward a comprehensive understanding of how to change beliefs. It suggests that a change in beliefs requires an integrated approach that seeks to build a new network, one that can grow strong enough to eventually feature a more efficient coupling than the old network. Thus, the network theory promises to substantially advance our understanding of belief change. To what extent are these theoretical analogies between ecosystems and beliefs substantiated analytically?

### Degree of Order in Self-Sustaining Networks vs. Mental Systems

Based on the conceptual understanding of ecosystems as self-sustaining networks, several measures have been derived to capture relevant properties of such systems, including degree of order, robustness, stability, and ascendancy (e.g., Ulanowicz, 1986, 2011; Jørgensen and Ulanowicz, 2009). Could the same measures capture relevant properties of belief data? The argument is that a valid extension of a network-based measure, should we find support for it, could also support the extension of conceptual claims. In other words, if a measure derived from the idea of self-sustaining networks manages to capture relevant features of beliefs, then we conclude that beliefs, too, can be conceptualized as self-sustaining networks. This is arguably an indirect way of seeking support for a conceptual leap. Nevertheless, it is a first step toward a conceptualization of beliefs as networks, one that would justify the development of more direct measures down the line. In what follows we focus on a measure of order.

#### How to Conceptualize Order in a Self-sustaining Network?

**FIGURE 2:**
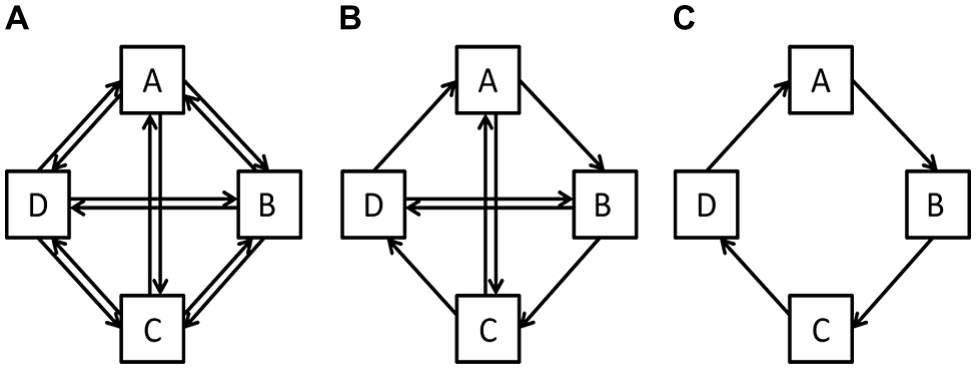
**Schematic of three idealized self-sustaining networks to illustrate the different degrees to which energy/meaning is coupled among components. (A)** Highly redundant system (yielding disorder). **(C)** Highly efficient system (yielding order). **(B)** System with intermediate recycling efficiency (adapted from Ulanowicz, 2000).

To address this question, consider the schematic networks represented in **Figure 2**. The networks differ in the way in which their components are connected to each other: at one extreme (**Figure 2A**), each component is linked with all other components, yielding high redundancy; and at the other extreme (**Figure 2C**), each component is linked with only one component, yielding low redundancy. The middle figure shows a configuration in which there is some redundancy in component connections while there are also some missing connections. Importantly, the network is most ordered when redundancy of connections is lowest, and when constraints on connections are highest.

Applied to ecosystems, where components are individual species and the connection is energy flow, **Figure 2A** shows a system in which energy is flowing in all directions. A good example is the ecosystem of a rainforest climate, where there is high redundancy in how energy is being exchanged between species. For such a system to sustain itself, a lot of external energy is necessary, as there is very little recycling of energy. In contrast, **Figure 2C** shows an ecosystem that is highly constrained in energy flow, one that recycles sufficient energy to survive strong fluctuations in external energy (e.g., a mountain climate ecosystem). In this latter system, the redundancy is very low, making the system vulnerable to changes in individual species. The ecosystem shown in **Figure 2B** is intermediate in redundancy of energy flow, allowing for some recycling of energy as well as some flexibility, to accommodate disruptions in individual species.

Applied to a belief, where meaning is being entrained, components are individual experiences, and a connection is the coupling among similarities, **Figure 2A** shows a case in which various similarities are coupled. Experience A might be coupled with Experience B on one feature, and Experience B might be coupled with Experience A on another feature. The result is an unorganized network of experiences, where various similarities are each amplified a little. Such a network needs continuous external support on what feature to pay attention to, as the network does not provide sufficient guidance. In contrast, **Figure 2C** shows a belief in which the coupling is highly constrained. Experience A is coupled with Experience B on one feature, and the same feature is coupled between Experiences B and C. This case is highly ordered, the coupling of similarities being highly constrained. The belief shown in **Figure 2B** is intermediate in the degree to which similarity between experiences is constrained.

Using these insights, the degree of order can be calculated using information theory (cf. Ulanowicz, 1986, 1998, 2000, 2009; Szyrmer and Ulanowicz, 1987; Lerner, 2006). Specifically, there are two terms of interest, that of *uncertainty* (*H*) and that of *average mutual information* (*AMI*). Uncertainty *H* is a measure that depicts the amount of “unknown” that exists in a distribution of scores (Shannon and Weaver, 1949)^2^. The uncertainty of an outcome can be estimated when a specific cue has previously occurred. If all possible states of a system have the same probability, then the system has the maximum uncertainty. If the outcome can adopt only one state, then the system has the minimum uncertainty. Mathematically, the equation is:

$$H=-K\Sigma p_{i}\,\log_{2}p_{i},$$

where *p_i_* is the probability of a score (and *K* is a constant set to 1). If the probabilities are evenly distributed across the possible scores, uncertainty is maximal.

Average mutual information is conceptualized as a structure or a quantifiable pattern that leads to a reduction in uncertainty. It measures the level of articulation among the components, based on distribution of scores (Castillo and Kloos, 2013). Put differently, *AMI* reflects the decrease in uncertainty that results from observation of past outcomes of similar or associated events. Mathematically, *AMI* is a logarithmic correlation between two distributions that can be estimated by using the conditional probability of a_j_ given b_i_. Specifically, its formula is:

$$AMI=k\underset{i,j}{\Sigma }p(a_{i},b_{j})\log\left[\frac{p(b_{i}|a_{j})}{p(b_{i})}\right],$$

where *k* is a constant set to 1, *p*(a_j_, b_i_) is the joint probability of events in two separate distributions, *p*(b_i_) is the probability of the variable b_i_, and *p*(b_i_|a_j_) is the conditional probability of b_i_ given a_j_.

Finally, a compound measure between *AMI* and *H* reflects the degree of order (*α*), an indicator of the constraints on the connections between components. The degree of order is a measure of organization and structure. In formal terms, the degree of order can be represented as the ratio between *AMI* and its respective uncertainty *H*:

α=AMI/H

If degree of order is close to zero in an ecosystem, the recycled energy is too low to deal with perturbations in external energy flow. This system is unlikely to possess sufficient cohesion to maintain its integrity and identity over time. It fits the network shown in **Figure 2A**, where there is no unique energy flow. In this hypothetical case, the network has highest levels of uncertainty and lowest levels of AMI. In contrast, if *α* is high in an ecosystem, close to 1, the system recycles energy efficiently, but is vulnerable if a chain gets disrupted or a component gets perturbed. It fits the network shown in **Figure 2C**, where it is possible to precisely predict the direction of flow. Here, the network has lowest levels of uncertainty and highest levels of AMI. **Figure 2B** represents a network in which degree of order adopts intermediate values, given that the flow has some uncertainty as well as some constraints.

#### Do Macroscopic Measures of Order Map onto the Order in Predictions?

In what follows, we address this question using accuracy data from a group of participants who were asked to make guesses about how transparent containers behave in water^3^. Reasoning about sinking objects is typically attributed to incorrect beliefs (e.g., Inhelder and Piaget, 1958; Smith et al., 1985; Kohn, 1993; Kloos and Somerville, 2001; Kloos, 2007). Thus, this domain lends itself to studying the degree of order in predictions, especially in the context of corrective feedback.

**FIGURE 3:**
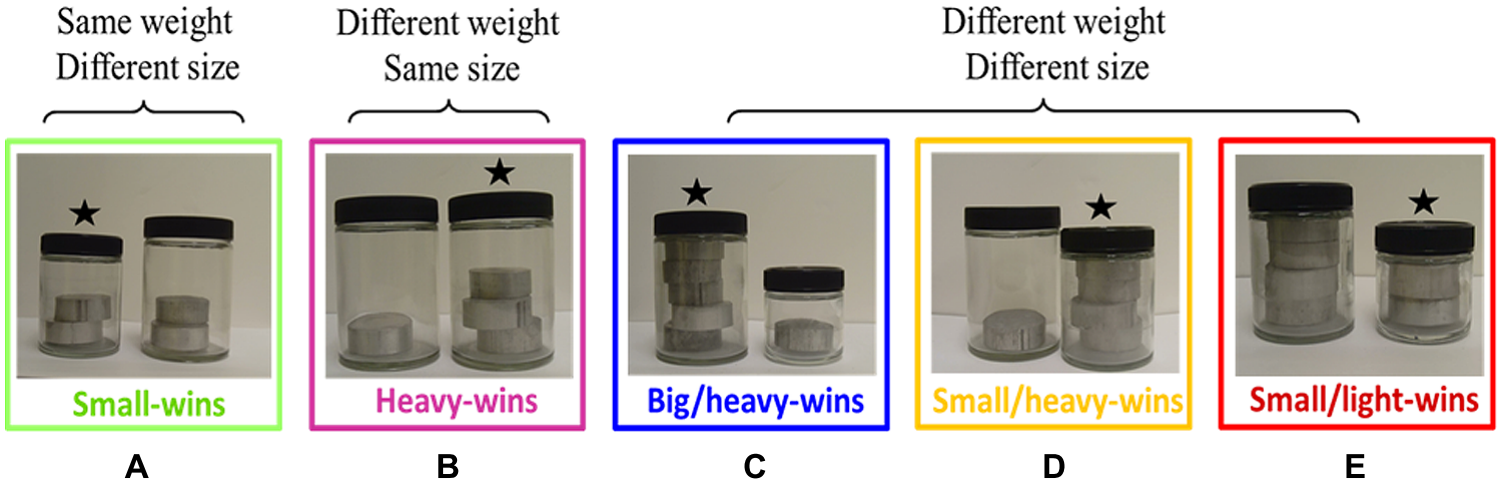
**Example pairs of objects, one for each different trial type.** The object that sinks faster in the pair is marked with a star. **(A)** Small-wins pair; **(B)** heavy-wins pair; **(C)** big/heavy-wins pair; **(D)** small/heavy-wins pair; **(E)** small/light-wins pair.

What follows is a brief outline of the experiment (see Appendix A for a full description of the method). The specific task was to predict which of two objects would sink either faster (sink-faster condition) or slower (sink-slower condition). Objects were transparent containers of different sizes that could hold a certain number of aluminum disks. They were paired up such that it was sometimes the heavier and sometimes the lighter object that sank faster. For example, in the small-wins trial type, both objects had equal mass and the smaller one sank faster. **Figure 3** shows the five possible trial types.

**FIGURE 4:**
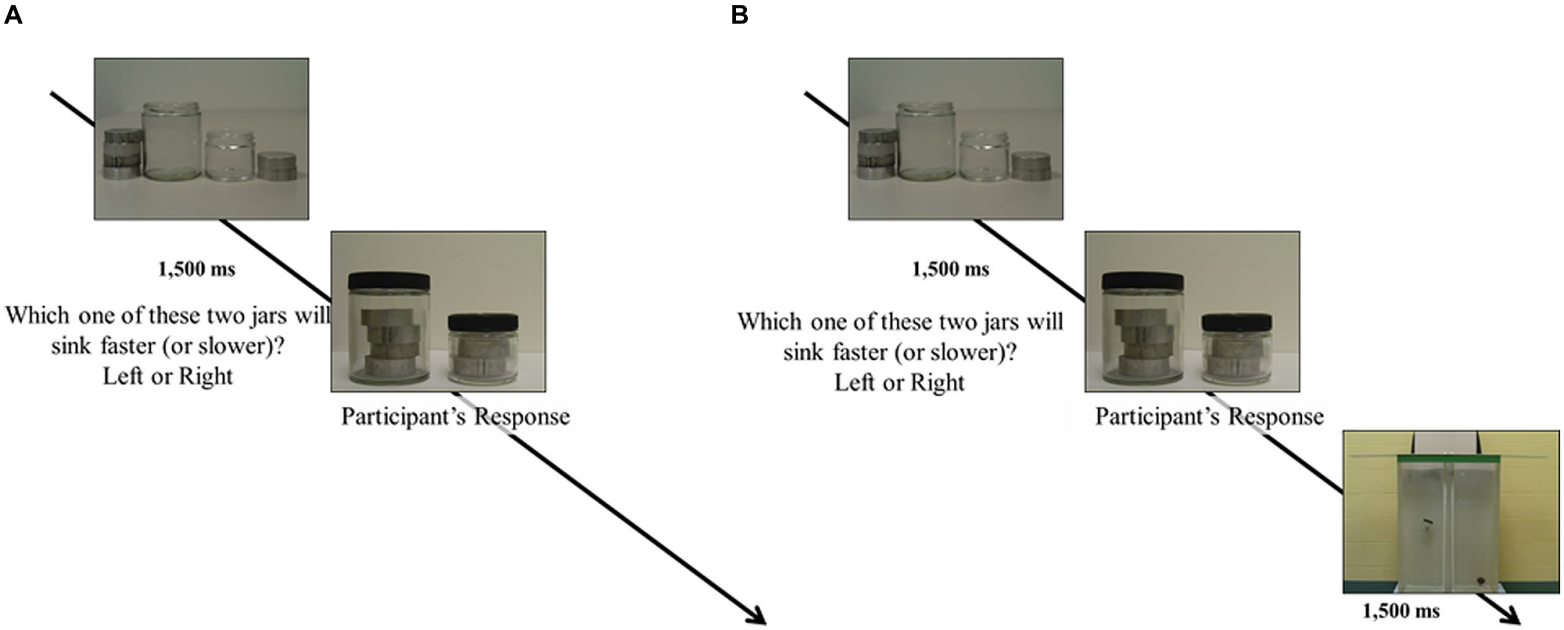
**Schematic representation of prediction trials. (A)** Prediction trial without feedback. **(B)** With feedback (a picture is shown of the objects after being dropped in a tank of water).

There were a total of 360 trials, broken up into eight segments of 45 trials each. The first two segments (Pre1 and Pre2), as well as the last two segments (Post1 and Post2), were simple prediction trials. In contrast, the middle four segments (T1, T2, T3, and T4) contained feedback, provided after each prediction. **Figure 4** shows a schematic of the procedure during each kind of trial. We anticipated that the small changes in instructions (between groups) and the presence of feedback (within groups) would generate a different pattern of performance, based on existing findings (Waldmann, 2001; cf. Fernbach et al., 2010; Öllinger et al., 2012; Kloos and Sloutsky, 2013). If the proposed measure of degree of order is reliable, it should track these changes, independently of the different patterns that the specific instructions and feedback elicit.

Group data (separated by condition, type of trial and segment) were analyzed in three different ways. First, we analyzed the proportion of correct predictions, to shed light on participants’ understanding of the task, the effect of the training, and whether the specific instruction had an effect on performance. Second, we estimated the degree of uncertainty in different types of trials, the AMI between these groups, and the resulting degree of order across segments. Finally, the same data set was subjected to a *non-linear categorical principal components analysis* (CATPCA). This type of analysis differs from standard PCA in that the relations between variables are assumed to be non-linear (as well as linear), rather than exclusively linear^4^. CATPCA (i.e., has been applied to behavioral data previously, and therefore could serve as a reasonable comparison to our new approach (Linting et al., 2007). The main analysis then pertains to a comparison between degree of order and the proportion of variance in the data explained by the first dimension extracted through CATPCA.

Note that all of these analyses are based on group data, which is nothing unusual for a discussion of accuracy. It nevertheless carries an important caveat, especially in the realm of the study of beliefs. This is because a participant’s performance on one trial is not independent of the participant’s performance on a next trial. Trial-by-trial performance is instead connected by a belief, a belief that might differ for different participants. Collapsing across participants masks these details, and thus cannot speak to the question of how beliefs emerge or change for individual participants. We use group data nevertheless because it allows us to investigate whether the measure of order developed for ecosystems (degree of order) tracks the changes in order observed in a group of participants who made predictions about sinking objects (proportion of explained variance).

##### Accuracy

**FIGURE 5:**
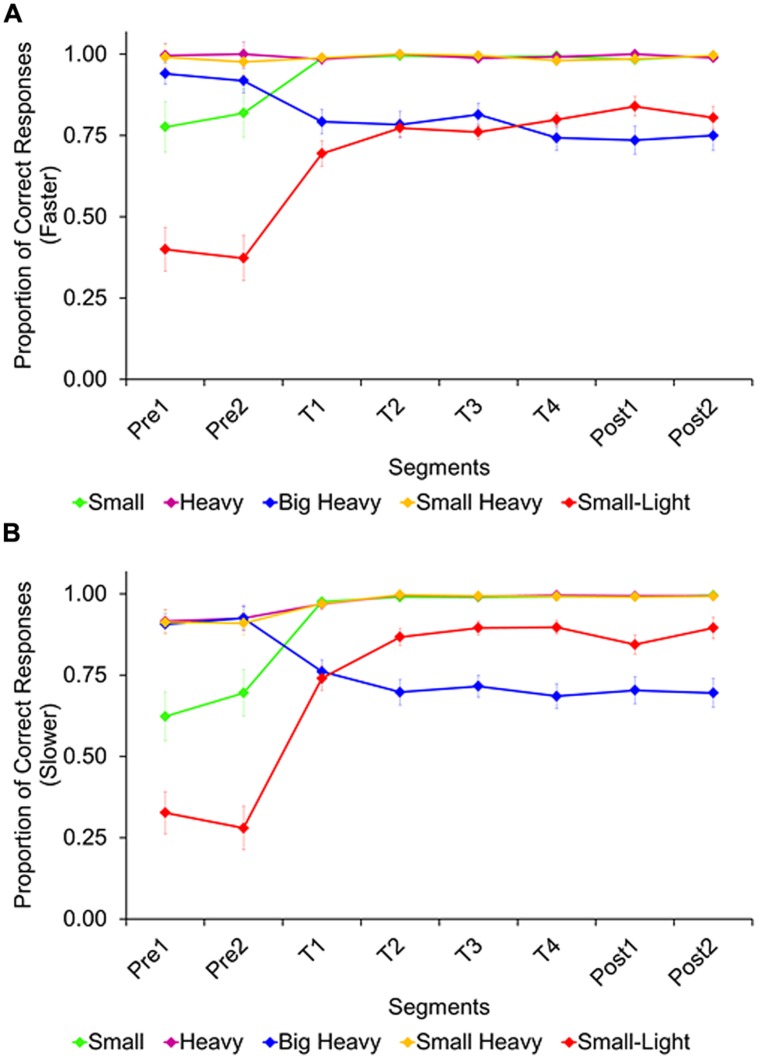
**Average proportion of correct responses, by trial type and segment, in the sink-faster **(A)** and the sink-slower condition **(B)**.** Error bars represent the standard errors.

In the sink-faster condition (**Figure 5A**), there was a significant linear increase of accuracy across segments, *F*(7,168) = 10.62, *p* < 0.001, $\eta_{p}^{2}$ = 0.31, with lower accuracy during the first two segments (*M*_Pre1_ = 0.82, *M*_Pre2_ = 0.82) than during the subsequent segments (*M*_T1_ = 0.89, *M*_T2_ = 0.91, *M*_T3_ = 0.91, *M*_T4_ = 0.90, *M*_Post1_ = 0.91, *M*_Post2_ = 0.91), *ps* < 0.01. A significant interaction between trial type and segment, *F*(28,672) = 14.05, *p* < 0.01, $\eta_{p}^{2}$ = 0.37, shows that this linear increase in accuracy was present for the small-wins and the small/light-wins trial types, *p*s < 0.01, but not for the other trial types. In fact, there was a linear decrease in accuracy for the big/heavy-wins trial type, *p* < 0.01.

In the sink-slower condition (**Figure 5B**), there was also a significant linear increase of accuracy across experimental segments, *F*(7,182) = 18.84, *p* < 0.01, $\eta_{p}^{2}$ = 0.42, with lower accuracy during the first two segments (*M*_Pre1_ = 0.74, *M*_Pre2_ = 0.75) than during the subsequent segments (*M*_T1_ = 0.88, *M*_T2_ = 0.91, *M*_T3_ = 0.92, *M*_T4_ = 0.91, *M*_Post1_ = 0.91, *M*_Post2_ = 0.92), *ps* < 0.01. As in the other condition, we again found a significant interaction between trial type and segment, *F*(28,728) = 20.39, *p* < 0.001, $\eta_{p}^{2}$ = 0.44, showing that the linear increase in accuracy was present for the small-wins and the small/light-wins trial types, *p*s < 0.01, but not for the other trial types. Again, there was a linear decrease in accuracy for the big/heavy-wins trial type, *p* < 0.01.

The two conditions were compared in a mixed-design ANOVA (trial type vs. condition vs. segment). While there was no difference between condition, *F*(1,50) = 1.48, *p* = 0.23 (*M*_sink-faster_ = 0.88 vs. *M*_sink-slower_ = 0.87), there was a marginally significant interaction between trial type and condition, *F*(4,200) = 2.08, *p* = 0.08, $\eta_{p}^{2}$ = 0.04. This interaction was driven by the big/heavy-wins and small/light-wins trial types, visible most clearly after the initial two segments without feedback, at the onset of feedback trials and afterward. Specifically, while participants in the sink-faster condition performed similarly high in the two trial types once feedback was provided, *p* > 0.99, participants in the sink-slower condition performed better in the small/light-wins than the big/heavy-wins trials, *ps* < 0.05. Thus, they were more likely to pick the bigger/heavier object as the slower sinker.

Taken together, participants responded correctly when the heavier object sank fastest. When the smaller object sank faster, response accuracy was close to chance or even below chance. Despite feedback, overall accuracy did not reach ceiling (i.e., predicting all trials correctly in the later segments). In fact, as accuracy increased on one trial type, it decreased on another trial type. Even so, participants modified their performance under feedback, and variations in the wording (faster vs. slower) generated different performances.

##### Measures derived from ecosystem analyses

As mentioned above, the three measures of interest here pertain to uncertainty, AMI, and degree of order. Findings for each of these measures are presented in turn.

Uncertainty (*H*) was estimated for each segment, across participants. The first step was to transform the raw data into quartiles, based on the distribution of scores of a trial type in each segment of a condition. Specifically, a participant’s average accuracy in a trial type was replaced by one of four values, representing its quartile in the distribution of scores. This was necessary to make data comparable across different trials. The quartile data were then used to create contingency tables for each pair of trial types of each segment in a condition. Finally, we converted each contingency table into a joint-probability table, by dividing each entry by the total number of participants (see Appendix B for an illustrative example calculation).

**FIGURE 6:**
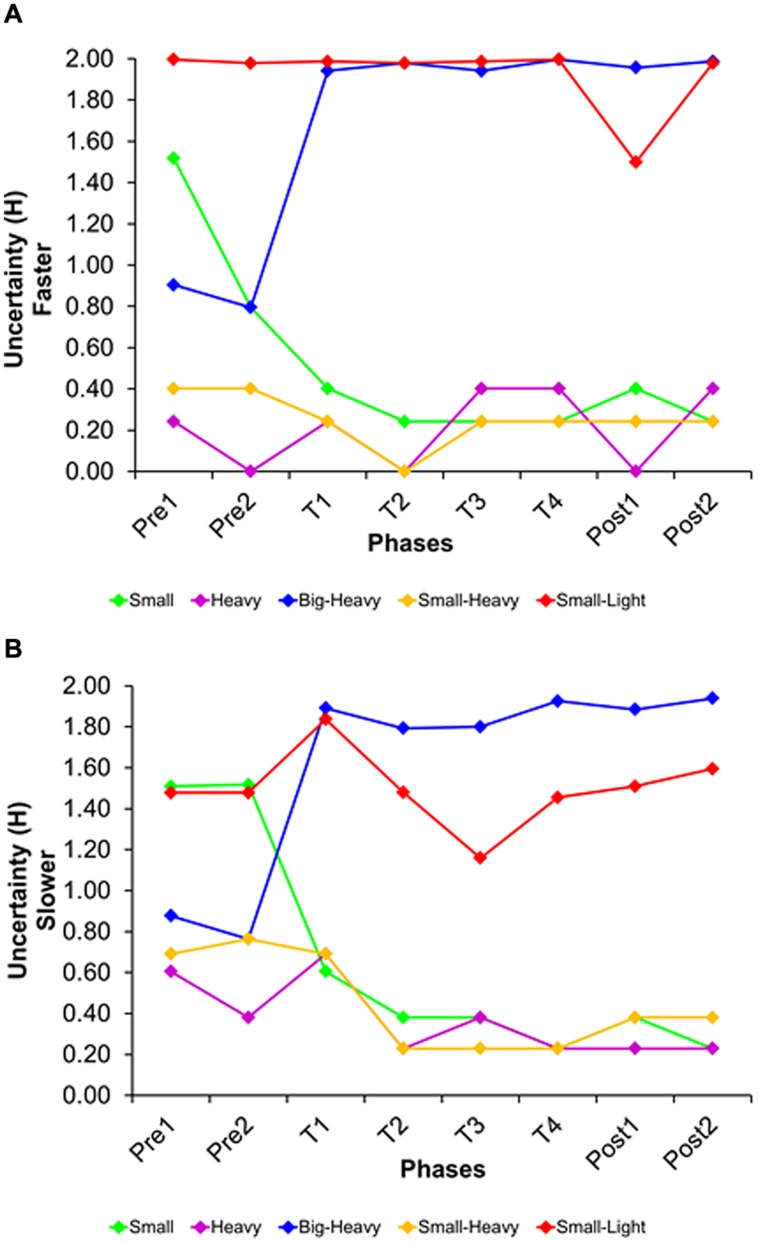
**Uncertainty *H* of each trial type, separated by segment and trial type, in the sink-faster **(A)** and the sink-slower condition **(B)****.

The second step was to use the marginal probabilities of the joint-probability matrix to estimate uncertainty for a specific segment and trial type (following Eq. 2; see Appendix B for the necessary steps in these calculations). The resulting uncertainty for each trial type is shown in **Figure 6**, separated by segment and condition. Note that uncertainty was highest for the small/light-wins trial type, almost reaching *H_max_* = 2 in some segments. The trial type big/heavy-wins also moved toward increased uncertainty over time. While it was relatively low at first, it came close to maximal uncertainty during feedback trials and then stayed there. On the other end of the spectrum, the trial types small/heavy-wins and heavy-wins had low uncertainty at the beginning, with little change across segments. Uncertainty of the small-wins trial type started out relatively high, but then dropped once feedback was given.

Average mutual information captures the amount of reduced uncertainty in the responses on one trial type that results from information about responses to another trial type. To calculate the *AMI* for each directional pair of trial types, we first estimated the conditional probability *p*(b_i_|a_j_) that a participant’s performance in one trial type would be in one quartile (*a_j_*) given that the participant’s performance on anther trial type is in a certain quartile (*b_i_*). We then calculated the *AMI* for each directional pair of trial types, following Eq. 2. Appendix B shows a step-by-step description of the estimation of *AMI* between two trial types.

**FIGURE 7:**
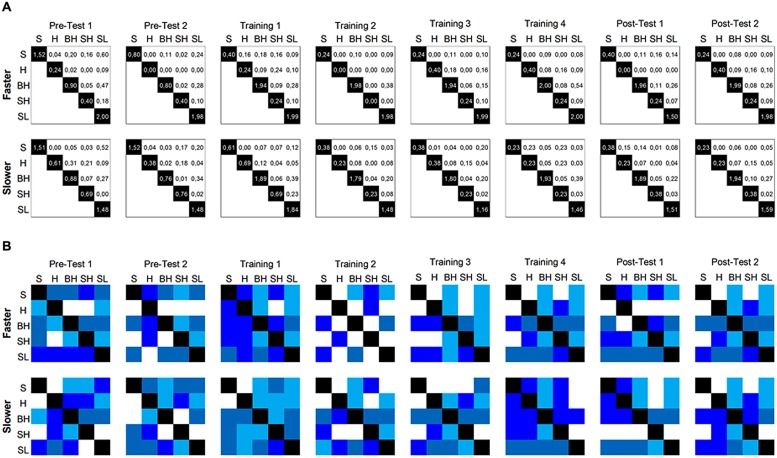
**Average mutual information **(A)** and degree of order **(B)** obtained for each trial-type pair, separated by segment and condition [the diagonal in **(A)** shows *H* of each trial type].** The shading represents a quartile of degree of order (white: lowest level; blue: highest level). S, small-wins pair; H, heavy-wins pair; BH, big/heavy-wins pair; SH, small/heavy-wins pair; SL, small/light-wins pair.

The resulting *AMI*s for each directional pair are shown in **Figure 7A**. Note that *AMI* tended to be highest for the link between the small/light-wins and small-wins trial types, suggesting that performance in these two trial types was interrelated. Similarly, there was a connection between the trial types small/light-wins and big/heavy-wins. Thus, predictions on trials in which the smaller and lighter object sank fastest tended to be coupled with predictions on trials in which either the smaller object sank fastest or the bigger and heavier object sank fastest. This pattern of performance cannot be explained by mere fluctuations in accuracy, nor can it be explained by mere fluctuations in uncertainty. Thus, fluctuations in *AMI* capture a unique aspect of the data.

**FIGURE 8:**
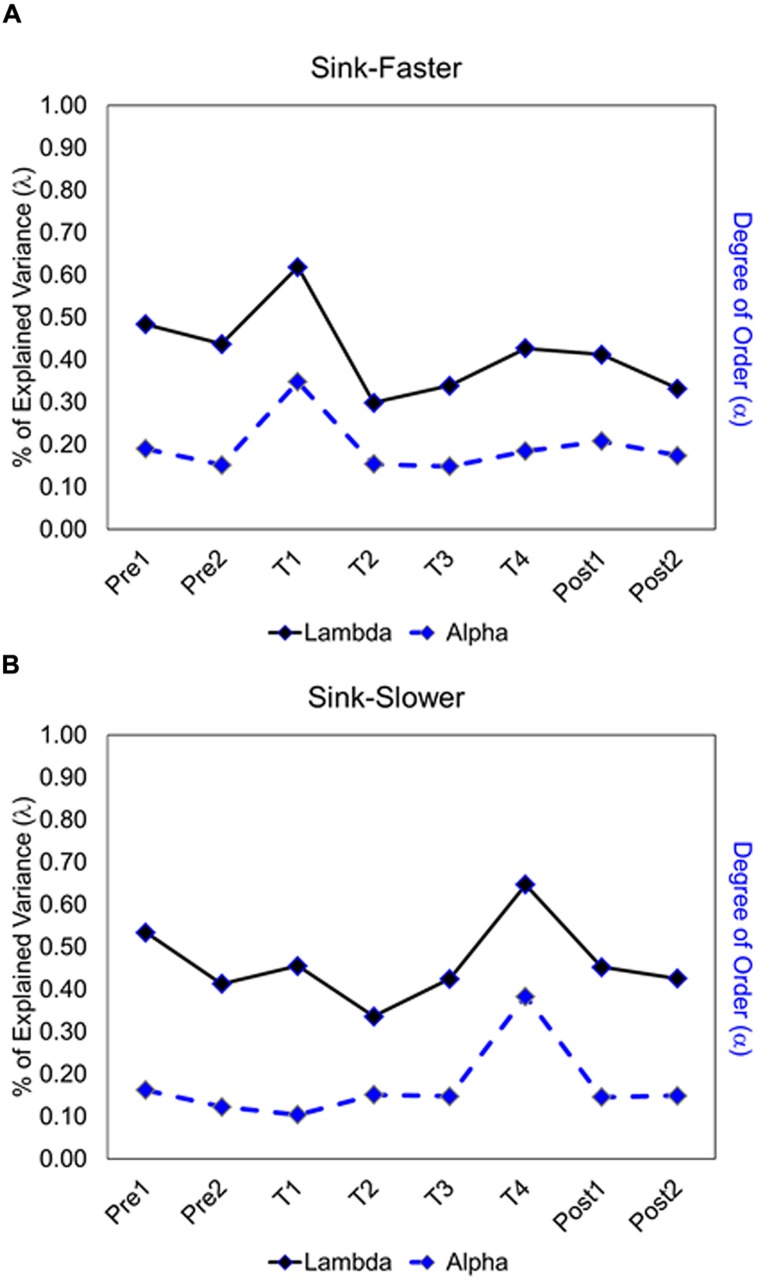
**Fluctuations of degree of order and % of explained variance of the first dimension by segment in the sink-faster **(A)** and the sink-slower condition **(B)****.

The degree of order (*α*) is estimated as the ratio between *AMI* and its respective trial type uncertainty *H* (see Eq. 3), capturing the articulation between pairs of trial types. **Figure 7B** shows the results, with the absolute size of *α* being represented as quartile (dark blue is indicative of the highest degree of order). Note that the values below and above the diagonal are different because *AMI* values (of pairs of trial types) are divided by the *H* of different trial types. Results show a strong fluctuation in degree of order for different pairs of trial types, as well as a strong fluctuation across segments. This latter fluctuation is captured in **Figure 8**, displaying the average degree of order obtained for a given segment (see dashed line). Specifically, there was a spike in degree of order at the onset of the feedback trials in the sink-faster condition (**Figure 8A**), and a spike in degree of order at the end of the feedback trials in the sink-slower condition (**Figure 8B**).

Importantly, these spikes in degree of order could not have been anticipated by the fluctuation in accuracy. Thus, our analysis of order in adults’ predictions is by no means redundant with what we could have gotten from an analysis of accuracy. It provides a novel way of capturing relevant patterns in performance, one that might shed light on underlying processes beyond what accuracy measures could not. Drawing again on the analogy of ecosystems, nobody would be surprised to learn that self-sustaining networks are not necessarily accurate. To quote Lorenz (1963/2002, p. 260), “To the biologist (…) it is in no way surprising to find (…) some details [that] are unnecessary or even detrimental to survival.” Self-sustaining networks need not to be adaptive in the way veridical systems are (cf. Kelty-Stephen and Eddy, 2015). The fluctuations of order found in adults’ predictions, orthogonal to accuracy, underscore this point.

##### Categorical principal component analysis

The CATPCA extracts dimensions from the correlation pattern among trial types to represent the degree to which they are associated with each other (Linting et al., 2007). In the case in which trial types are uncorrelated, the number of dimensions returned by the CATPCA is equal to the number of trial types. In contrast, if trial types have shared variability, then the number of dimensions decreases. The smaller number of dimensions indicates that the network components are coupled, and they have a common structure that is articulating their functionality. The eigenvalue of a dimension, *03BB*, represents the amount of variability in the correlation pattern that can be explained by that dimension. **Figure 8** shows the two measures, *03B1* and *03BB*, side by side, as they change over time.

##### Relation between degree of order and proportion of explained variance

To what extent does the degree of order track the proportion of explained variance? To answer this question, we calculated the correlation between the average degree of order and the eigenvalue of the first dimension extracted by CATPCA. The fluctuations, including the spikes, match with the variations observed in the proportion of explained variance for the first dimension. A positive correlation between these two measures was reliably detected (sink-faster, *r* = 0.85, *p* = 0.007; sink-slower, *r* = 0.80, *p* = 0.017). This convergent finding is a criterion of validation, because two structural measures of order, applied at different times during the same learning process, capture ordered patterns of decision.

These findings show that the measure developed to capture network stability closely tracked the proportion of explained variance obtained by CATPCA. Thus, we found evidence that the measure used in information theory to capture network order is strongly correlated with fluctuations in traditional measures of order. Our findings lend support to the idea that beliefs could be understood as self-sustaining networks of experiences.

## Conclusion

In this paper, we have presented an approach to beliefs that is motivated by insights about the organization of ecosystems. We define beliefs in terms of self-sustaining networks, characterized by processes of autocatalysis, circular causality, and centripetality. In support of these claims, we found in a behavioral dataset that the changes in degree of order were captured in CATPCA: an increase in degree of order was significantly correlated with an increase in the proportion of explained variance. The particular measure applied here, degree of order, allows us to leave behind questions about the exact content of stable structure. This index incorporates the informational measures of *H* and *AMI* that focus on the system organization, not the details of the components that make it up. Thus, degree of order makes it possible to incorporate the heterogeneity of mental structures into explanations of cognitive phenomena without trapping us in a discussion of what exactly it is that humans know or do not know.

Our network approach to beliefs departs significantly from the prevalent conception of beliefs as a collection of propositions. At its center lies the idea that individual experiences become coupled with each other, in a way that is both constraining and strengthening. Thus, beliefs – like ecosystems – are neither a direct reflection of an invariant property in the actor-environment relation, nor are they completely separate from it. Order comes instead from a coordination that amplifies itself under the right conditions. This conceptualization throws overboard the reliance on reductionism, and it allows for moment-to-moment dynamics, non-linear changes, and the emergence of something entirely new.

There are several important advantages to the network view of beliefs. First, they fit the data on beliefs: that they often emerge spontaneously, that they affect subsequent experiences, and that they are difficult to change. Second, they fit within the larger theory of how self-organizing processes give rise to stability, thus paving the way for progress that is not tied to the specific material content of a system. And finally, our approach circumvents the dead-end that has plagued approaches that seek linear causality and reductionist explanations.

It remains to be seen whether the principles of self-sustaining networks apply to scales of order that are above and below that of a single belief. For example, beliefs that are organized into mental models or theories (e.g., Johnson-Laird, 1983) might be networks themselves, with individual beliefs serving as nested components in the organization (i.e., systems-of-systems of self-sustaining networks). And on the other end of the hierarchy of order, it is possible that even a single experience – a representation – is a network as well (cf. Byrge et al., 2014). If so, then the idea of representations is misleading: our mind does not represent the environment any more than the flow of energy in an ecosystem represents the distribution of energy in the environment.

## Conflict of Interest Statement

The authors declare that the research was conducted in the absence of any commercial or financial relationships that could be construed as a potential conflict of interest.

## Footnotes

1 We propose that the coupling happens on the basis of a match between experiences. It is clear that a match alone cannot explain the emergence of the coupling; additional forces are needed to explain the basis for the network. We will return to this issue in Part 2.

2 This measure is also referred to as entropy in the literature (Ulanowicz, 1986).

3 All experimental methods reported here were approved by the university’s IRB to ensure that they are in line with ethical guidelines for research with human subjects.

4 In linear PCS all variables are scaled at the interval or ratio numeric level of measurement. In non-linear PCA, by constrast, such assumptions are not necessary, because correlations are not computed between the observed variables but between the quantified variables. Indeed, the correlation matrix in non-linear PCA is not fixed; it is dependent on the type of quantification, the level of analysis being chosen for each of the variables. Thus the non-linear PCA output is not obtained from the correlation matrix. It is iteratively calculated from the raw data, using the optimal scaling procedure to quantify the variables according to their analysis level.
