# Supplementary material for: Beliefs as Self-Sustaining Networks: Drawing Parallels Between Networks of Ecosystems and Adults’ Predictions
Source: Front Psychol. 2015 Nov 12;6:1723. doi: 10.3389/fpsyg.2015.01723 (PMC4641980; doi:10.3389/fpsyg.2015.01723)
Supplement: Supplementary file 1 [file Data_Sheet_1.PDF]

Appendix A: Method of the Experiment

Participants. Participants (N = 52) were recruited from the pool of undergraduate students enrolled in an introductory psychology course at a large Midwestern university. They received partial course credit for their participation in one of two conditions. For the sink-faster condition, there were 14 women and 11 men, and for the sink-slower condition, there were 12 women and 15 men, ranging in age from 18 to 28 years (M = 20.78 years, SD = 2.37).

Materials. Real-life sinking objects were used to generate pictorial stimuli. They were transparent glass jars that differed in their sizes: Large jars were 8 cm high and 6.3 cm wide; medium jars were 6.9 cm high and 5.8 cm wide, and small jars were 5 cm high and 5.3 cm wide. Round aluminum discs (1 cm high, 4 cm in diameter, 43 g) could be placed inside the jars to manipulate mass. There were 12 size/disc combinations, depending on the jar's size and the number of weights inside the jar. The jars were combined into pairs of objects, resulting in five different trial types (depending on how mass and volume correlated with rate of sinking).

Figure 3 shows an example for each trial type, the faster sinking objects being marked with a star. In two of the trial types, only one of the features was varied (either mass or volume), and in three of the trial types, both mass and volume were varied. Specifically, in the *small-wins* pair (Figure 3A), mass was held constant and the size of jar was varied in such a way that the smaller jar sank faster. In the *heavy-wins* pair (Figure 3B), volume was held constant and mass was varied in such a way that the heavier jar sank faster. In the *big/heavy-wins* pair (Figure 3C), the faster sinking object was bigger and heavier than the slower object. In the *small/heavy-wins* pair (Figure 3D), the faster sinking object was smaller and heavier than the slower object. And finally, in the *small/light-wins* pair (Figure 3E), the faster sinking object was smaller and lighter than the slower object. Note that mass and volume correlate positively for both the big/heavy-wins pair type and the small/light-wins pair type. Thus, these two types cannot be discriminated on the basis of how mass correlates with volume. Yet, even though the two types of pairs cannot be distinguished ahead of time, the outcome of the race differs by type: The faster sinking object is big and heavy in some cases and not in others.

There were nine pairs of each trial type, resulting in a total of 45 unique pairs. Each unique pair was photographed, with alternating left-right position of the jars, and with discs inside as well as outside of the jars (see Figure 4). To obtain feedback, each unique pair was dropped in a 1 m tall water tank to create videos of the sinking objects. A dividing wall separated the water tank into two sides (each with a floor area of 1 x 1 ft^2). This made it possible for each jar to sink without being affected by the turbulences made by the other object. Pictures of sinking objects were created in bitmap format, using stills from the video recordings of the jars being dropped in the tank of water. We also created close-up pictures of pairs of objects, used for participants' predictions. A numeric keypad was used to record participants' predictions.

Procedure. Participants were tested individually in the lab, using DirectRT Precision Timing Software (Version 2012) to administer the experiment on a desktop computer. The experiment consisted of a familiarization phase and eight prediction phases. In the sink-faster condition, the task was to predict which of the two objects would sink faster. And in the sink-slower condition, the task was to predict which of the two objects would sink slower.

During familiarization, participants were first shown the empty jars, as well as a series of aluminum discs. They were told that all the discs have the same weight. The experimenter then filled the large and small jars with aluminum discs and asked the participant to predict which of them would sink faster in water (or slower, depending on the condition). Participants were encouraged to lift the jars before making their predictions. Then they were provided with feedback. Finally, participants were shown the keypad and how it works. Prior to the experiment proper, they were informed that the pictures shown in the experiment were taken from the real objects.

The prediction trials started immediately after familiarization. For each trial, participants were first shown an image of two empty jars next to each other, with a stack of discs by each jar. This allowed participants a clear view of the number of discs for each object. After 1.5 seconds, the image was replaced with one that shows the same two jars, but now filled with the discs and closed with a lid. Participants were asked to decide which of the two jars would sink faster (or slower). There was no time restriction for making a prediction. Trials without feedback ended when the participant provided a prediction, pressing the keypad to indicate the choice. Trials with feedback, by contrast, showed the feedback picture right after a prediction was made, advancing after it had been shown for 1.5 seconds. The feedback picture showed which jar had reached the bottom of the tank first. On the very first feedback trial, the image was explained. Figure 4 illustrates the method in a given trial (either with or without feedback).

In total, there were 360 prediction trials, yielding eight segments of 45 trials each. The first two segments served as a pre-test (Pre1 and Pre2): participants made their predictions across various jar combinations, without receiving any feedback. The next four segments served as training (T1, T2, T3, and T4): predictions were followed by corrective feedback. Finally, the last two segments served as a post-test (Post1 and Post2), featuring trials that were identical to the initial two segments.

Appendix B. Illustrative Calculations of *H*, *AMI*, and *α*

Table B.1

Illustrative Example Contingency Table and Resulting Joint-Probability Table

Contingency Table

		Trial Type (a): Small/light-wins				
		Q1	Q2	Q3	Q4	b_i
Trial Type (b): Small-wins	Q1	5	1	1	0	7
	Q2	1	2	3	0	6
	Q3	0	3	3	6	12
	Q4	0	0	0	0	0
a_i		6	6	7	6	25

Joint-Probability Table $p(a_j, b_i)$

		Trial Type (a): Small/light-wins				
		Q1	Q2	Q3	Q4	$p(b_i)$
Trial Type (b): Small-wins	Q1	0.20	0.04	0.04	0.00	0.28
	Q2	0.04	0.08	0.12	0.00	0.24
	Q3	0.00	0.12	0.12	0.24	0.48
	Q4	0.00	0.00	0.00	0.00	0.00
$p(a_j)$		0.24	0.24	0.28	0.24	1.00

Note. Data were obtained for two trial types in the first segment of the sink-faster condition (small/light wins; small-wins). Quartiles are shown as Q1-Q4. Data are represented as number of participants per pair of quartiles. Marginals show the number of participants per quartile for a trial type (a_j; b_i). As a way of reference, note that the performance of 5 of the 25 participants was in the first quartile for both trial types. And the joint probability $p(a_j, b_i)$ of the 5 participants located in the first quartile of both trial types corresponds to a probability of 0.20, given that $p(a_{Q1}, b_{Q1})$ = 5/25 = 0.20.

Table B.2

Illustrative Calculation of Uncertainty (H) using Data from Table B.1.

	Trial Type (b): Small-wins			Trial Type (a): Small/light-wins		
Quartile	p_i	$-\log_2 p_i$	$-p_i * \log_2 p_i$	p_i	$-\log_2 p_i$	$-p_i * \log_2 p_i$
Q1	0.280	-1.837	-0.514	0.240	-2.059	-0.494
Q2	0.240	-2.059	-0.494	0.240	-2.059	-0.494
Q3	0.480	-1.059	-0.508	0.280	-1.837	-0.514
Q4	--	--	--	0.240	-2.059	-0.494
		H_b:	1.516		H_a:	1.996

Note. For each trial type, p_i represents the marginal probability. Q1-Q4 represent the quartiles of the trial-type distribution, and $-log_2\, p_i$ is the uncertainty associated with a specific quartile. Resulting uncertainties are shown in the shaded area. They are both smaller than H_{max}, and $H_a > H_b$, suggesting that there was more uncertainty associated with the small/light-wins trial type than with the small-wins trial type.

Table B.3

Conditional-Probability Tables using Data from Table B.1.

	$p(a_j \mid b_i)$				$p(b_i \mid a_j)$			
	Trial Type (b): Small-wins				Trial Type (a): Small/light-wins			
Quartile	Q1	Q2	Q3	Q4	Q1	Q2	Q3	Q4
Q1	0.83	0.17	0.14	0.00	0.71	0.14	0.14	0.00
Q2	0.17	0.33	0.43	0.00	0.17	0.33	0.50	0.00
Q3	0.00	0.50	0.43	1.00	0.00	0.25	0.25	0.50
Q4	0.00	0.00	0.00	0.00	0.25	0.25	0.25	0.25

Note. As a way of reference, the conditional probability of b_{Q1}, given a_{Q1}, in the small-wins trial type is equal to 0.83, given that $p(b_{Q1} \mid a_{Q1}) = p(a_{Q1} \cap b_{Q1}) / p(a_{Q1}) = (5/25) / (6/25) = 0.83$

Table B.4

Calculation of Average Mutual Information using Data from Tables B.1 and B.3.

i, j	$p(a_j, b_i)$	$p(b_i\|a_j)$	$p(b_i)$	$p(b_i\|a_j) / p(b_i)$	$p(a_j, b_i) * \log_2 [p(b_i\|a_j) / p(b_i)]$
1,1	0.20	0.83	0.28	2.98	0.31
1,2	0.04	0.17	0.28	0.60	-0.03
1,3	0.04	0.14	0.28	0.51	-0.04
1,4	0.00	0.00	0.28	0.00	0.00
2,1	0.04	0.17	0.24	0.69	-0.02
2,2	0.08	0.33	0.24	1.39	0.04
2,3	0.12	0.43	0.24	1.79	0.10
2,4	0.00	0.00	0.24	0.00	0.00
3,1	0.00	0.00	0.48	0.00	0.00
3,2	0.12	0.50	0.48	1.04	0.01
3,3	0.12	0.43	0.48	0.89	-0.02
3,4	0.24	1.00	0.48	2.08	0.25
4,1	0.00	0.00	0.00	1.04	0.00
4,2	0.00	0.00	0.00	1.04	0.00
4,3	0.00	0.00	0.00	0.89	0.00
4,4	0.00	0.00	0.00	1.04	0.00
		Total *AMI*			0.60

Note. Here (i, j) represent the different quartiles of small/light-wins trial type (j) and small-wins trial type (i), $p(a_j, b_i)$ is the joint probability of each possible pair of quartiles, $p(b_i)$ is the marginal probability of small-wins trial type (see also Table B.1), and $p(b_i|a_j)$ is the conditional probability calculated in Table B.3.

Table B.5

AMI Obtained for each Pair of Trial Types from the First Segment of the Sink-faster Condition

	S	H	BH	SH	SL
S	1.52	0.04	0.20	0.16	0.60
H		0.24	0.02	0.00	0.09
BH			0.90	0.05	0.47
SH				0.40	0.18
SL					2.00

Note. Uncertainty (*H*) of each trial type appears in the grey area. S: small-wins; H: heavy-wins; BH: big/heavy-wins; SH: small/heavy-wins; SL: small/light-wins. As a way of reference, note that the AMI for the SL/S combination is the same value we have calculated in Table B.4.

Table B.6

Degree of order (α) based on Data from Table B.5

	S	H	BH	SH	SL
S		0.180	0.226	0.399	0.303
H	0.029		0.025	0.012	0.043
BH	0.135	0.094		0.116	0.237
SH	0.106	0.020	0.052		0.091
SL	0.399	0.356	0.522	0.452	

Note. Trial types are represented by capital letters. S: small-wins; H: heavy-wins; BH: big/heavy-wins; SH: small/heavy-wins; SL: small/light-wins.
